# Heavy metal accumulation and its association with epididymal sperm morphometrics in dromedary camels with penile and preputial pathologies

**DOI:** 10.14202/vetworld.2025.2867-2877

**Published:** 2025-09-30

**Authors:** Montaser Elsayed Ali, Ahmed Yassen M. Osman, Hassan A. Hussein, Mohammed A. Alqahtani, Maha Abdullah Momenah, Ahmed Ezzat Ahmed, Ragab Hassan Mohamed

**Affiliations:** 1 Department of Animal Productions, Faculty of Agriculture, Al-Azhar University, 71524 Assiut, Egypt; 2 Department of Theriogenology, Faculty of Veterinary Medicine, Aswan University, Aswan 81528, Egypt; 3 Department of Theriogenology, Faculty of Veterinary Medicine, Assiut University, Assiut, 71526, Egypt; 4 Department of Surgery, Obstetrics and Artificial Insemination, Faculty of Veterinary Medicine, Sphinx University, New Assiut, Egypt; 5 Department of Biology, College of Science, King Khalid University, 61413 Abha, Saudi Arabia; 6 Department of Biology, College of Science, Princess Nourah bint Abdulrahman University, Riyadh 11671, Saudi Arabia; 7 Prince Sultan Bin Abdelaziz for Environmental Research and Natural Resources Sustainability Center, King Khalid University, Abha 61421, Saudi Arabia

**Keywords:** aluminum, dromedary camels, heavy metals, nickel, penile pathology, sperm morphometry, zinc

## Abstract

**Background and Aim:**

Sperm morphometry is a vital indicator of fertility, yet male germ cells are highly vulnerable to environmental toxins such as heavy metals. Dromedary camels (DCs) are frequently exposed to penile and preputial pathologies, conditions that can impair semen quality. This study aimed to assess epididymal sperm morphometric characteristics in camels with genital lesions and to explore their associations with concentrations of aluminum (Al), zinc (Zn), molybdenum (Mo), nickel (Ni), and lead (Pb).

**Materials and Methods:**

Sixty adult male DCs (8–10 years, n = 10/group) were classified into six groups: injury-free, balanoposthitis, penile trauma, prolapsed prepuce, phimosis, and penile tumors. Blood samples were analyzed for heavy metal concentrations using inductively coupled plasma mass spectrometry. Epididymal spermatozoa (caput, corpus, cauda) were evaluated for sperm length (SL), sperm head length (SHL), sperm head width (SHW), sperm head length-to-width ratio (SHL/W), and sperm tail length. Sperm abnormalities were categorized into proximal/distal cytoplasmic droplets and tail defects. Data were analyzed by analysis of variance and correlation statistics.

**Results:**

Sperm morphometrics varied significantly with pathological status. Camels with penile and preputial disorders exhibited increased SHW in caudal spermatozoa, while SHL and SHW were reduced in caput spermatozoa (p < 0.01). The phimosis group showed fewer abnormalities and lower total abnormal sperm, whereas the penile tumor group had elevated proximal and distal defects and the highest abnormality rate. Heavy metal analysis revealed significantly increased Zn and Ni concentrations in the penile tumor group, while Mo and Pb levels showed no significant differences. Correlation analysis indicated strong associations of Al and Zn with SHW and SHL/W in caudal spermatozoa, and Ni with SHL, SHW, and SHL/W in corpus spermatozoa (p < 0.05).

**Conclusion:**

Epididymal sperm morphometrics are influenced by penile and preputial pathologies and are significantly associated with Al, Zn, and Ni concentrations. These findings highlight the potential role of heavy metal accumulation in camel reproductive dysfunction and emphasize the need for monitoring environmental exposure in breeding regions.

## INTRODUCTION

Dromedary camels (DCs) represent approximately 95% of the global camel population and are predominantly distributed across Africa, the Middle East, and Asia, where they play an essential socioeconomic role in arid regions [1]. The Arabian camel, or one-humped camel, is particularly common in northern Africa, with populations estimated at 9.4 million in Chad, 4.9 million in Sudan, and nearly 99,000 in Egypt, according to FAOSTAT reports [1, 2]. Owing to their aggressive mating behavior and susceptibility to transport-related trauma, DCs are especially prone to penile and preputial disorders that can compromise semen quality [3].

Balanoposthitis is an inflammatory disorder of the penis and prepuce, clinically manifested by pruritus, discharge, erythema, and rash [4]. Penile trauma typically involves rupture of the tunica albuginea, a fibrous sheath enclosing the corpora cavernosa, and is often referred to as a penile fracture, usually resulting from accidental injury [5, 6]. Prolapse of the prepuce denotes a reversible inversion of the foreskin, leading to exposure of the internal mucosal layer on the external surface [7, 8]. Phimosis arises when the foreskin fails to retract over the glans, thereby narrowing the penile opening [9, 10]. Penile tumors, usually presenting as small, painless lesions, may also cause paraphimosis, which prevents foreskin retraction [11].

Male germ cells are among the most vulnerable tissues to heavy metal toxicity [12]. Zinc (Zn) deficiency has been linked to impaired spermatogenesis, reduced libido, and oxidative damage to testicular cells [13, 14]. Elevated molybdenum (Mo) exposure has been shown to cause dose-dependent reductions in sperm concentration and an increase in sperm abnormalities [15]. Even low to moderate exposure to lead (Pb) adversely affects reproductive parameters and contributes to sperm DNA fragmentation [16, 17].

Aluminum (Al) has been reported to impair sperm motility, morphology, and concentration, whereas the effects of nickel (Ni) remain less conclusive [18]. Both Al and Ni can induce structural changes in the testes and epididymis and disrupt trace element interactions [19]. Zn is essential for sperm capacitation and the acrosome reaction, while Pb interferes with the initiation and progression of the acrosome reaction [20]. In camels, heavy metals tend to accumulate in tissues such as blood, liver, meat, and rumen fluid due to environmental exposure, metabolism, and dietary intake [21]. Their concentrations vary widely depending on geography, seasonal fluctuations, soil composition, and water sources [22].

Although heavy metals are well recognized as reproductive toxicants in humans and other mammals, their specific impact on sperm morphometry and quality in DCs remains poorly characterized. Most previous studies have concentrated on gross semen traits or testicular histopathology, whereas detailed investigations of epididymal sperm morphometrics (caput, corpus, and cauda) are scarce. Furthermore, despite the frequent occurrence of penile and preputial disorders in camels–often resulting from aggressive mating behavior and transport-related trauma–very few studies have examined how these pathological conditions interact with heavy metal accumulation to influence sperm structure. Abdelrahman *et al*. [23] reviewed the trace element status in camels and reported substantial variations in blood mineral concentrations depending on geography, season, and diet. However, they also highlighted the lack of mechanistic studies linking trace element levels to reproductive pathologies and sperm abnormalities. While individual effects of elements such as Zn, Al, and Ni on male fertility have been described, their correlation with epididymal sperm morphometrics under pathological conditions in camels is still unexplored. This gap limits understanding of the multifactorial drivers of reproductive inefficiency in camels and constrains the development of effective diagnostic and preventive strategies for herd fertility management.

This study aimed to investigate the associations between heavy metal concentrations and epididymal sperm morphometrics in DCs affected by penile and preputial pathologies. Specifically, the objectives were to: (i) quantify the concentrations of Al, Zn, Mo, Ni, and Pb in the blood of camels with different genital pathologies; (ii) evaluate morphometric parameters of epididymal spermatozoa, including sperm length (SL), sperm head length (SHL), sperm head width (SHW), sperm head length-to-width ratio (SHL/W), and sperm tail length (STL) across epididymal segments; (iii) assess the incidence of abnormal sperm forms under various pathological conditions; and (iv) analyze correlations between heavy metal concentrations and sperm morphometrics in diseased versus injury-free camels. By integrating pathological classification with elemental profiling and morphometric analysis, this research provides novel insights into the role of environmental toxicants in camel reproductive dysfunction and establishes a foundation for improved herd fertility management in regions where camels are of major socioeconomic importance.

## MATERIALS AND METHODS

### Ethical approval and informed consent

The authors confirm that the ethical policies of the journal, as noted on the journal’s author guidelines page, have been adhered to. This study was conducted in accordance with the Institutional and National Guidelines for the care and use of animals, which were followed according to the World Organization for Animal Health (WOAH) standards. Ethics approval from an Institutional Review Board (IRB) has been granted by the Research Ethics Committee; Reference No: 01-2025-00015. Informed consent was obtained from all owners before study inclusion.

### Study period and location

The study was conducted from December 2023 and September 2024 in the Aswan Governorate, Egypt (24°05′20′′N, 32°53′59′′E), located on the eastern bank of the Nile River, approximately 900 km South of Cairo. The animals used were privately owned by farmers in Draw, Kom Ombo, Edfu, and Abu Sunbul.

### Experimental grouping and pathological diagnosis

A total of 60 clinically mature male DCs (*Camelus dromedarius*), aged 8–10 years, with body condition scores between 2.5 and 3.5 and an average body weight of 425 ± 85 kg, were included. Camels with penile and preputial disorders were physically restrained in a supine position using ropes on the limbs and a halter on the head. A morphological examination was performed to confirm normal testicular status.

Animals were categorized into six groups (n = 10 each) based on penile and preputial conditions: Injury-free (control), balanoposthitis, penile trauma, prolapsed prepuce, phimosis, and penile tumors. The classification of disease cases in this study was based on the definitions, as shown in Table 1 [4, 10, 24]. All animals were raised primarily on open pasture. Alfalfa (*Medicago sativa*) was provided when pasture was insufficient, and water was available *ad libitum*.

**Table 1:** Grouping of the pathological problems of the penis and prepuce in male dromedary camels.

Pathological problems	Identification	References
Balanoposthitis	Inflammation refers to various conditions affecting the penis and prepuce, such as penile pain, pruritus, discharge, erythema, rash, or inconsolable sobbing.	[4]
Penile trauma	Traumatic rupture of the tunica albuginea, the fibrous covering of the two cylinders, known as corpora cavernosa, which runs along the penis, is also known as a traumatic rupture.	
Prolapsed prepuce	The reversible turning of the foreskin inside out is called prolapse of the prepuce, denoting the appearance of one layer of internal skin covering the outside layer.	[10]
Phimosis	A condition where the penis opening narrows due to the foreskin retracting behind the glans, the skin retracts inability (foreskin or prepuce) covering the penis head (glans).	
Penile tumors	Genital warts are small tumors on the genital area. Penile tumors present as painless lumps on the penis that can prevent foreskin retraction.	[24]

### Sample collection and transport

#### Sample acquisition

Camels were slaughtered at the Aswan Municipal Slaughterhouse, and testes were collected immediately post-slaughter within 3–5 min of exsanguination. A total of 120 epididymal samples (6 groups × 10 animals × 2 testes) were obtained. The sample size was determined based on preliminary data and power analysis (power = 0.80, α = 0.05).

#### Sample transport

The epididymides were carefully dissected, cleared of surrounding tissues, and placed in 50 mL Falcon tubes (BD Falcon, USA) containing 25 mL of pre-warmed (37°C ± 0.5°C) 0.9% saline solution (NaCl, #S7653, Sigma-Aldrich, USA). Samples were transported to the laboratory within 60 ± 5 min, with constant temperature monitoring maintained using a calibrated digital thermometer (accuracy ±0.1°C; Traceable Control Company, Massachusetts, USA).

#### Laboratory processing

Laboratory surfaces were disinfected with 70% ethanol and maintained at 22°C ± 1°C. Instruments were autoclaved at 121°C for 20 min and inspected for sterility using normal saline. The epididymal cauda, corpus, and caput were separated and longitudinally incised using sterile blades (No. 10, Swann-Morton, UK). Spermatozoa were flushed out, washed 3–4 times in Brackett and Oliphant medium, and maintained at 37°C [25].

#### Sperm morphometric analysis

Smears were prepared by mixing 5 μL of semen with eosin-nigrosin stain (5% eosin, bluish and 10% nigrosin, 1:4 ratio) and applying it onto pre-warmed (36°C) slides. Slides were air-dried at room temperature. A total of 120 slides (6 groups × 10 animals × 2 slides) were examined.

Following World Health Organization guidelines, 30 μL semen aliquots were placed on Leja 4-chamber slides (20 μm depth; International Medical Veterinary, L’Aigle, France) and assessed under a fluorescence microscope (400×, Cat. No. 9126000, Cypress, California, USA). Spermatozoa with stained heads were considered non-viable, whereas unstained heads were viable.

Morphometric evaluation included: SL, SHL, SHW, SHL/W, and STL.

Abnormal spermatozoa were categorized as proximal cytoplasmic droplet (PCD), distal cytoplasmic droplet (DCD), bent tails, coiled tails, or folded tails (Dag defects).

### Biochemical assays

#### Blood sampling and inductively coupled plasma mass spectrometry (ICP-MS) analysis

Blood samples (n = 60; 10/group) were collected before slaughter. Serum was separated by centrifugation (3,000 × *g*, 20 min) and stored at −20°C. Elemental concentrations of Al, Zn, Mo, Ni, and Pb were determined using ICP-MS (6500 Duo, Thermo Scientific, UK) following Nakaguchi *et al*. [26].

#### Sample preparation

Serum was thawed, diluted 1:10 in 2% ultrapure nitric acid (HNO_3_; TraceSELECT Ultra, MilliporeSigma, Burlington, Massachusetts, USA), and filtered through 0.45 μm polytetrafluoroethylene syringe filters (MilliporeSigma, MA, USA), analogous to 0.2 μm polycarbonate filtration methods for dissolved metals.

#### Instrument calibration

Calibration curves (0.5–100 μg/L) were prepared using certified multi-element standards (Merck, Germany). Internal standards were included as per high resolution-ICP-MS protocols.

#### Accuracy and precision

Recovery rates ranged between 90% and 110%, with intra- and inter-day precision below 5% relative standard deviation. limit of detection was: Al, 0.02 μg/L; Zn, 0.05 μg/L; Ni, 0.005 μg/L; Pb, 0.002 μg/L [27].

### Statistical analysis

Data were analyzed using Statistical Package for Social Sciences v25 (IBM, Chicago, USA). Normality was tested with the Kolmogorov–Smirnov test. One-way analysis of variance was applied, followed by Duncan’s multiple range test for *post hoc* comparisons. The statistical model used was:

Yij = μ + Ti + Aj + eij

Where:

- Yij = Observed value
- μ = Overall mean
- Ti = Effect of treatment group (pathological vs. normal)
- eij = Random error term].

## RESULTS

### Normal and abnormal epididymal sperm

As presented in Table 2, sperm morphometric parameters, including SL, SHL, SHW, SHL/W, and STL from the cauda, corpus, and caput regions of camels with penile and preputial pathologies, did not differ significantly (p > 0.05) from those of the injury-free group. Abnormal spermatozoa exhibiting morphological defects such as PCD, DCD, or tail abnormalities (bent, coiled, folded) were not assessed for SL and STL.

**Table 2:** Morphometrics of normal spermatozoa obtained from the cauda, corpus, and caput epididymidis of injury-free and pathologically injured dromedary camels.

Morphometric analysis of sperm (μm)	Injury-free	Pathological issues					SEM	p-value

		Balanoposthitis	Penile trauma	Prolapsed prepuce	Phimosis	Penile tumors		
Cauda epididymidis								
SL	49.36^ns^	46.90^ns^	49.79^ns^	47.72^ns^	50.97^ns^	47.81^ns^	0.84	>0.05
SHL	5.39^ns^	4.66^ns^	5.42^ns^	5.10^ns^	4.89^ns^	4.98^ns^	0.17	>0.05
SHW	2.77^ns^	3.11^ns^	3.33^ns^	2.62^ns^	2.57^ns^	2.93^ns^	0.19	>0.05
SHL/W	2.06^ns^	1.49^ns^	1.73^ns^	2.06^ns^	2.01^ns^	1.76^ns^	0.10	>0.05
STL	43.97^ns^	42.24^ns^	44.37^ns^	42.62^ns^	60.75^ns^	42.83^ns^	2.65	>0.05
Corpus epididymidis								
SL	45.93^ns^	45.96^ns^	45.97^ns^	45.20^ns^	44.25^ns^	47.30^ns^	1.23	>0.05
SHL	4.34^ns^	5.13^ns^	5.79^ns^	5.25^ns^	4.60^ns^	5.27^ns^	0.25	>0.05
SHW	3.55^ns^	2.91^ns^	3.24^ns^	3.19^ns^	3.24^ns^	3.28^ns^	0.18	>0.05
SHL/W	1.32^ns^	1.80^ns^	1.87^ns^	1.67^ns^	1.78^ns^	1.62^ns^	0.14	>0.05
STL	41.59^ns^	40.81^ns^	40.18^ns^	39.95^ns^	39.32^ns^	42.01^ns^	1.06	>0.05
Caput epididymidis								
SL	42.28^ns^	44.36^ns^	47.11^ns^	43.48^ns^	43.40^ns^	46.71^ns^	1.20	>0.05
SHL	5.76^ns^	5.06^ns^	5.73^ns^	5.16^ns^	6.36^ns^	5.79^ns^	0.22	>0.05
SHW	3.10^ns^	3.04^ns^	3.70^ns^	3.22^ns^	3.30^ns^	3.43^ns^	0.12	>0.05
SHL/W	1.95^ns^	1.66^ns^	1.56^ns^	1.62^ns^	2.02^ns^	1.72^ns^	0.10	>0.05
STL	36.53^ns^	39.30^ns^	41.38^ns^	38.32^ns^	37.04^ns^	40.92^ns^	1.20	>0.05

ns = Non significance, μm = Micrometer, SL = Sperm length, SHL = Sperm head length, SHW = Sperm head width, SHL/W = Sperm head length/width, STL = Sperm tail length, SEM = Standard error of the mean.

A notable exception was observed in the caudal epididymal spermatozoa, where SHW was significantly increased (p < 0.01) in camels with penile and preputial pathologies compared with the control group. No significant differences (p > 0.05) were detected in corpus epididymal sperm morphometrics between pathological and injury-free camels. However, spermatozoa from the caput epididymis exhibited significantly reduced SHL and SHW (p < 0.01) in camels with penile and preputial disorders compared with injury-free animals (Table 3).

**Table 3:** Morphometrics of the abnormal spermatozoa obtained from the injury-free cauda, corpus, and caput epididymidis and pathological issues of dromedary camels (means).

Morphometric analysis of sperm (μm)	Injury-free	Pathological issues					SEM	p-value

		Balanoposthitis	Penile trauma	Prolapsed prepuce	Phimosis	Penile tumors		
Cauda epididymidis								
SHL	4.84^ns^	5.03^ns^	5.52^ns^	5.53^ns^	5.09^ns^	4.84^ns^	0.15	>0.05
SHW	2.00^b^	2.34^a^	2.35^a^	2.55^a^	2.51^a^	2.58^a^	0.06	<0.05
SHL/W	2.50^ns^	2.18^ns^	2.37^ns^	2.18^ns^	2.03^ns^	1.88^ns^	0.08	>0.05
Corpus epididymidis								
SHL	4.70^ns^	4.85^ns^	5.01^ns^	4.57^ns^	4.77^ns^	5.25^ns^	0.11	>0.05
SHW	2.25^ns^	2.67^ns^	2.55^ns^	2.33^ns^	2.26^ns^	1.93^ns^	0.12	>0.05
SHL/W	1.75^ns^	1.82^ns^	2.00^ns^	2.08^ns^	2.36^ns^	3.52^ns^	0.24	>0.05
Caput epididymidis								
SHL	6.33^a^	4.20^b^	3.83^b^	3.85^b^	4.30^b^	4.47^b^	0.24	<0.01
SHW	3.94^a^	1.78^bc^	1.59^c^	1.28^c^	1.36^c^	2.71^b^	0.25	<0.01
SHL/W	1.36^c^	2.61^ab^	2.74^ab^	3.14^a^	3.26^a^	1.65^bc^	0.22	<0.05

^a,b,c^ = Duncan analyses, μm = Micrometer, different superscripts in the same row are significantly differ,

ns = Non significance, SL = Sperm length, SHL = Sperm head length, SHW = Sperm head width, SHL/W = Sperm head length/width, STL = Sperm tail length, SEM = Standard error of the mean.

### Morphometric analysis of abnormal epididymal spermatozoa

The influence of penile and preputial pathologies on abnormal spermatozoa is shown in Table 4. The frequency of PCDs in caudal epididymal spermatozoa was significantly higher (p < 0.01) in camels with penile trauma, penile tumors, and prolapsed prepuce compared with the control group. By contrast, the injury-free group exhibited the highest percentage of DCD, which was significantly reduced (p < 0.01) in all pathological groups.

**Table 4:** Sperm abnormalities in the cauda and corpus epididymidis obtained from the injury-free and pathological issues male dromedary (means).

Formation of sperm (μm)	Injury-free	Pathological issues					SEM	p-value

		Balanoposthitis	Penile trauma	Prolapsed prepuce	Phimosis	Penile tumors		
Cauda epididymidis								
PCD	24.00^a^	9.00^b^	29.00^a^	25.00^a^	7.00^b^	37.00^a^	2.71	<0.01
DCD	8.00^a^	3.00^b^	1.33^bc^	1.33^bc^	2.00^b^	1.67^bc^	0.67	<0.01
BST	7.00^c^	3.00^c^	13.00^b^	5.00^c^	17.00^a^	11.00^c^	1.25	<0.01
CST	4.00^a^	3.00^ab^	2.00^bc^	1.33^c^	1.67^bc^	1.00^c^	0.29	<0.01
Dag	15.00^a^	5.33^b^	3.33^b^	3.67^b^	1.33^b^	2.00^b^	1.20	<0.01
TAS	58.00^a^	51.00^a^	50.00^a^	43.00^b^	30.67^c^	53.33^a^	3.64	<0.01
TNS	42.00^c^	49.00^c^	50.00^c^	43.67^c^	68.00^b^	49.67^c^	4.09	<0.01
Corpus epididymidis								
PCD	15.67^bc^	26.67^b^	10.33^c^	23.00^b^	21.67^bc^	44.00^a^	2.86	<0.01
DCD	1.67^b^	5.00^a^	5.67^a^	3.67^a^	6.00^a^	4.67^a^	0.50	<0.05
PST	5.67^f^	10.67^cd^	13.67^bc^	16.33^b^	33.00^a^	9.67^d^	2.16	<0.01
CST	10.00^b^	2.67^c^	4.67^c^	17.67^a^	8.00^b^	3.00^c^	1.30	<0.01
Dag	5.00^abc^	5.67^ab^	1.33^c^	8.33^a^	3.00^bc^	2.67^c^	0.70	<0.05
TAS	44.00^ab^	62.00^a^	33.00^b^	50.00^ab^	43.00^ab^	63.00^a^	3.32	<0.05
TNS	56.00^ab^	38.00^c^	67.00^a^	45.00^bc^	54.67^ab^	39.00^c^	2.90	<0.05

^a,b,c,d,f^ = Duncan analyses, different superscripts in the same row are significantly differ, PCD = Proximal cytoplasmic droplet, DCD = Distal cytoplasmic droplet, Dag = Folded tail, TAS = Total abnormal spermatozoa, TNS = Total normal spermatozoa, BST = Bent of spermatozoa tail, CST = Coiled spermatozoa tail, SEM = Standard error of the mean.

Bent sperm tails were most common in the phimosis group, whereas coiled sperm tails (CST) were highest in the injury-free camels and lowest in those with penile tumors. Folded tail abnormalities (Dag defects) were significantly lower (p < 0.01) in all pathological groups compared with controls. Interestingly, the phimosis group demonstrated a significant reduction in total abnormal spermatozoa and a corresponding increase in total normal spermatozoa relative to the injury-free group. The highest CST frequency was recorded in the prolapsed prepuce group (p < 0.01). Overall, pathological groups exhibited higher DCD values, with the phimosis group showing the highest PCD incidence among all groups.

### Impact of heavy metals on epididymal spermatozoa

Concentrations of Al, Zn, Mo, Ni, and Pb under various pathological conditions are summarized in Table 5. Al levels were significantly higher (p < 0.01) in the prolapsed prepuce group compared with the control group, while the phimosis and penile tumor groups displayed intermediate levels. Zn concentrations were significantly elevated (p < 0.01) in camels with penile tumors, with moderate increases observed in the balanoposthitis and phimosis groups. In contrast, Mo levels showed no significant variation (p > 0.05) across groups. Ni concentrations were significantly higher (p < 0.05) in penile tumor cases compared with controls, whereas Pb concentrations did not differ significantly among groups.

**Table 5:** Minerals (mg/L): Al, Zn, Mo, Ni, and Pb concentrations in the injury-free and pathological issues of dromedary camels.

Mineral (mg/L)	Injury-free	Pathological issues					SEM	p-value

		Balanoposthitis	Penile trauma	Prolapsed prepuce	Phimosis	Penile tumors		
AL	34.71^b^	41.72^b^	40.29^b^	70.75^a^	58.79^ab^	51.47^ab^	4.08	<0.01
Zn	5.41^b^	9.49^ab^	3.97^b^	4.45^b^	10.34^ab^	21.42^a^	1.66	<0.01
Mo	1.61^ns^	0.78^ns^	0.71^ns^	3.99^ns^	2.57^ns^	3.21^ns^	0.51	>0.05
Ni	0.01^b^	0.02^ab^	0.01^b^	0.01^b^	0.01^b^	0.07^a^	0.01	<0.05
Pb	2.63^ns^	0.28^ns^	1.62^ns^	1.50^ns^	3.21^ns^	2.50^ns^	0.56	>0.05

^a,b,c^ = Duncan analyses,

ns = Non significance, Al = Aluminum, Zn = Zinc, Mo = Molybdenum, Ni = Nickel, Pb = Lead, SEM = Standard error of the mean.

### Correlation between heavy metals and sperm morphometrics

The correlation analysis is presented in Tables 6 and 7. No significant associations were found between Al, Zn, Mo, Ni, or Pb concentrations and the overall normality of spermatozoa from the cauda, corpus, or caput epididymal in pathological groups. However, in the caudal epididymal spermatozoa, Al concentrations were strongly and positively correlated with SHW (r = 0.630) and SHL/W ratio (r = 0.445). Moderate positive correlations were also observed between Zn levels and both SHW (r = 0.403) and SHL/W (r = 0.566).

**Table 6:** Correlation analysis among Al, Zn, Mo, Ni, and Pb concentrations and the normal sperm obtained from the epididymal in the injury-free and pathological issues of dromedary camels.

Items	Al		Zn		Mo		Ni		Pb	
				
	R-value	p-value	R-value	p-value	R-value	p-value	R-value	p-value	R-value	p-value
Cauda epididymidis										
SL	0.031	>0.05	0.056	>0.05	0.187	>0.05	0.266	>0.05	0.352	>0.05
SHL	0.272	>0.05	0.032	>0.05	0.134	>0.05	0.248	>0.05	0.173	>0.05
SHW	0.132	>0.05	0.155	>0.05	0.176	>0.05	0.168	>0.05	0.035	>0.05
SHL/W	0.027	>0.05	0.269	>0.05	0.160	>0.05	0.030	>0.05	0.129	>0.05
STL	0.186	>0.05	0.129	>0.05	0.111	>0.05	0.157	>0.05	0.088	>0.05
Corpus epididymidis										
SL	0.001	>0.05	0.148	>0.05	0.213	>0.05	0.193	>0.05	0.144	>0.05
SHL	0.319	>0.05	0.064	>0.05	0.294	>0.05	0.190	>0.05	0.010	>0.05
SHW	0.178	>0.05	0.052	>0.05	0.277	>0.05	0.001	>0.05	0.427	>0.05
SHL/W	0.344	>0.05	0.060	>0.05	0.073	>0.05	0.028	>0.05	0.320	>0.05
STL	0.084	>0.05	0.150	>0.05	0.176	>0.05	0.183	>0.05	0.153	>0.05
Caput epididymidis										
SL	0.087	>0.05	0.049	>0.05	0.210	>0.05	0.348	>0.05	0.266	>0.05
SHL	0.048	>0.05	0.050	>0.05	0.281	>0.05	0.218	>0.05	0.134	>0.05
SHW	0.014	>0.05	0.006	>0.05	0.032	>0.05	0.283	>0.05	0.118	>0.05
SHL/W	0.017	>0.05	0.074	>0.05	0.190	>0.05	0.0310	>0.05	0.206	>0.05
STL	0.095	>0.05	0.058	>0.05	0.261	>0.05	0.309	>0.05	0.290	>0.05

SL = Sperm length, SHL = Sperm head length, SHW = Sperm head width, SHL/W = Sperm head length/width, STL = Sperm tail length, Al = Aluminum, Zn = Zinc, Mo = Molybdenum, Ni = Nickel, Pb = Lead.

**Table 7:** Correlation analysis among Al, Zn, Mo, Ni, and Pb concentrations and the abnormal sperm obtained from the epididymal in the injury-free and pathological issues of dromedary camels.

Items	Al		Zn		Mo		Ni		Pb	
				
	R-value	p-value	R-value	p-value	R-value	p-value	R-value	p-value	R-value	p-value
Cauda epididymidis										
SHL	0.038	>0.05	0.378	>0.05	0.173	>0.05	0.046	>0.05	0.237	>0.05
SHW	0.630**	<0.01	0.403*	<0.05	0.232	>0.05	0.189	>0.05	0.020	>0.05
SHL/W	0.445*	<0.05	0.566*	<0.05	0.243	>0.05	0.090	>0.05	0.141	>0.05
Corpus epididymidis										
SHL	0.024	>0.05	0.168	>0.05	0.135	>0.05	0.546*	<0.05	0.204	>0.05
SHW	0.153	>0.05	0.141	>0.05	0.018	>0.05	0.522*	<0.05	0.225	>0.05
SHL/W	0.174	>0.05	0.285	>0.05	0.044	>0.05	0.761**	<0.01	0.076	>0.05
Caput epididymidis										
SHL	0.262	>0.05	0.055	>0.05	0.225	>0.05	0.045	>0.05	0.163	>0.05
SHW	0.456*	<0.05	0.079	>0.05	0.091	>0.05	0.266	>0.05	0.038	>0.05
SHL/W	0.471*	<0.05	0.205	>0.05	0.010	>0.05	0.360	>0.05	0.004	>0.05

* Correlation is significant at the 0.05 level,

** Correlation is significant at the 0.01 level. SL = Sperm length, SHL = Sperm head length, SHW = Sperm head width, SHL/W = Sperm head length/width, STL = Sperm tail length, Al = Aluminum, Zn = Zinc, Mo = Molybdenum, Ni = Nickel, Pb = Lead.

In the corpus epididymal, SHL, SHW, and SHL/W exhibited strong positive correlations with Ni concentrations (r = 0.546, 0.522, and 0.761, respectively). In the caput epididymal, Al concentrations showed moderate positive correlations with SHW (r = 0.546) and SHL/W (r = 0.471). Conversely, no significant correlations were observed between Mo or Pb concentrations and sperm morphometrics in any epididymal region of camels with penile and preputial pathologies.

## DISCUSSION

### Overview of findings

Male reproductive cells undergo a series of developmental transformations, starting with spermatogenesis in the seminiferous tubules and continuing through maturation in the epididymis [28]. This study is among the few to investigate the relationship between heavy metal concentrations, sperm morphometrics, and penile/preputial disorders in DCs. The findings provide novel insights into how pathological and environmental stressors influence reproductive performance. To explore these interactions, the concentrations of Al, Zn, Mo, Ni, and Pb were measured and correlated with epididymal sperm morphometry across pathological conditions.

### Impact of reproductive pathologies

Camels with penile and preputial disorders exhibited significantly increased SHW in caudal epididymal spermatozoa compared with injury-free controls. This suggests that the plasma membranes surrounding the sperm head and body may be less susceptible to damage than those associated with the tail and mitochondrial regions [29]. Conversely, SHL and SHW were significantly reduced in spermatozoa from the caput epididymis of pathological groups.

These findings align with earlier reports describing notable structural changes in sperm during spermatogenesis and epididymal maturation [30]. Testicular degeneration in camels with reproductive dysfunction often presents as disrupted spermatogenesis or Sertoli cell-only syndrome (SCOS) [31]. Prior studies also indicate that camels suffering from reproductive abnormalities exhibit reduced sperm motility and viability along with a high incidence of teratospermia [19, 28]. Collectively, these results confirm that reproductive pathologies are closely associated with complex sperm defects and testicular abnormalities, which negatively affect herd fertility.

### Effects of heavy metals on sperm morphology

Al concentrations were elevated in camels with prolapsed prepuce, with intermediate levels observed in phimosis and penile tumor groups. Al is known to impair sperm motility, morphology, and concentration, whereas the effects of Ni on sperm remain less conclusive [32]. Both Al and Ni were significantly associated with morphometric parameters of caudal epididymal spermatozoa, particularly SHW and SHL/W ratios. These metals are known to cause structural damage to testes and epididymis and disrupt trace element interactions [19, 33].

Zn concentrations were significantly higher in penile tumor cases. As the second most abundant trace element in blood after iron, Zn plays a crucial role in epithelial integrity, reproductive tract stability, sperm capacitation, and acrosome reaction [8, 34, 35]. Zn deficiency has been associated with impaired spermatogenesis, sperm abnormalities, and reduced testosterone levels [34]. In contrast, Ni exposure has been shown to damage testicular tissues and disturb trace element balance [36]. Pb disrupts acrosome reaction processes [20, 37], although in this study, Pb levels did not significantly vary across groups. Interestingly, Mo concentrations also showed no significant differences, despite prior evidence linking Mo exposure to reproductive toxicity [38]. Overall, these results suggest that oxidative stress and inflammatory mechanisms mediated by heavy metals, particularly Al, Ni, and Zn, likely contribute to reproductive disorders in camels, while Zn appears to be a protective factor in male fertility.

### Morphometrics of epididymal spermatozoa under pathological conditions

Analysis of sperm abnormalities revealed distinctive patterns linked to reproductive disorders. Increased frequencies of PCDs in penile trauma, penile tumor, and prolapsed prepuce groups indicate defective sperm maturation, as PCDs are typically eliminated during epididymal transit [39]. The concurrent reduction in DCD in these groups supports this interpretation, since retained PCDs are linked to impaired motility and reduced fertility [40, 41].

This study also found reduced DCDs and Dag defects in camels with penile and preputial pathologies compared with controls. Severe reproductive dysfunction, including azoospermia, has been linked to impaired Leydig cell function and altered sperm plasma membrane integrity [42]. The most severe sperm abnormalities were observed in the penile tumor group. Such defects are consistent with pathological conditions, including orchitis and cryptorchidism, which are known to impair spermatogenesis [31]. Furthermore, severe trauma may trigger testicular degeneration, SCOS, and spermatogenic arrest [43]. Interestingly, a high proportion of morphologically abnormal spermatozoa was also observed in clinically unaffected camels, suggesting possible subclinical spermatogenic dysfunction or intrinsic sperm quality defects [44, 45].

### Interpretation of correlation findings

Correlation analysis confirmed strong associations between heavy metal concentrations and sperm morphometrics. In particular, Al, Zn, and Ni were linked with SHW and SHL/W ratios across epididymal regions. Al showed moderate correlations with SHW and SHL/W in caput spermatozoa, suggesting interference with sperm maturation during early epididymal transit [46]. Ni exhibited strong correlations with SHL, SHW, and SHL/W in corpus spermatozoa, indicating a role in mid-epididymal morphogenesis.

These findings are consistent with earlier evidence that heavy metal exposure induces oxidative stress, leading to DNA fragmentation, protein damage, and reduced sperm motility and viability [47–49]. Both Al and Ni have been associated with structural changes in reproductive tissues, while Pb is known to impair sperm function and testosterone synthesis [50]. Accumulation of these metals may also compromise penile and preputial tissue integrity, thereby exacerbating pathological lesions [51].

## CONCLUSION

This study demonstrated that penile and preputial pathologies in DCs are associated with significant alterations in epididymal sperm morphometrics and are influenced by the accumulation of specific heavy metals. Notably, SHW was significantly increased in caudal spermatozoa of pathological groups, whereas SHL and SHW were reduced in caput spermatozoa compared with injury-free controls. Among pathological groups, camels with phimosis exhibited lower rates of sperm abnormalities, whereas penile tumors were linked to a higher incidence of proximal and distal defects, bent tails, and overall abnormal spermatozoa. Elemental profiling revealed significantly elevated concentrations of Zn and Ni in penile tumor cases, while Al was markedly higher in prolapsed prepuce cases. Correlation analyses confirmed strong associations between Al, Zn, and Ni concentrations and sperm morphometrics, particularly SHW and SHL/W ratios, highlighting their potential role in sperm structural integrity.

The findings underscore the importance of monitoring trace element exposure and reproductive health in camel herds. Given the socioeconomic value of camels in arid regions, integrating sperm morphometric assessment with heavy metal profiling may enhance reproductive management, facilitate early detection of subfertility, and optimize herd fertility. Preventive strategies, such as improved environmental monitoring, dietary adjustments, and early clinical intervention for penile and preputial disorders, could mitigate fertility losses in breeding programs.

A key strength of this study lies in its comprehensive design, which integrated pathological classification, detailed morphometric analysis of epididymal spermatozoa, and precise quantification of heavy metals using ICP-MS. By simultaneously considering multiple epididymal regions (caput, corpus, cauda), the study provides region-specific insights into sperm abnormalities and their association with environmental contaminants.

Despite its strengths, the study had limitations. The sample size, although statistically adequate, was limited to a single geographical region, which may limit its generalizability. Environmental exposure to heavy metals was not directly quantified in soil, water, or feed, which could have provided a broader ecological perspective. Furthermore, functional fertility outcomes, such as conception rates, were not assessed.

Future studies should expand to larger populations across diverse ecological zones to account for geographic and seasonal variability in heavy metal exposure. Investigations should also integrate oxidative stress biomarkers, hormonal assays, and molecular markers of sperm DNA integrity to elucidate the mechanistic pathways linking heavy metals with reproductive dysfunction. Intervention trials exploring dietary supplementation or antioxidant therapies could offer practical solutions to mitigate the adverse effects of heavy metals on camel fertility.

In conclusion, epididymal sperm morphometrics in DCs are strongly influenced by penile and preputial pathologies and by the accumulation of Al, Zn, and Ni. These findings provide novel insights into the interplay between environmental contaminants and reproductive health, laying a foundation for improved diagnostic, preventive, and management strategies in camel breeding systems.

## AUTHORS’ CONTRIBUTIONS

HAH, RHM, AYMO, and MEA: Prepared the conception and design of the study to perform data curation, blood sampling, and interpretation of data, statistically analyzed the data, and drafted the manuscript. MAM, MAA, and AEA: Investigation, statistical analysis, and edited the manuscript. All authors have read and approved the final version of the manuscript.
